# Nuclear envelope laminopathies: evidence for developmentally inappropriate chromatin-nuclear envelope interactions

**DOI:** 10.1186/1756-8935-6-S1-P65

**Published:** 2013-03-18

**Authors:** Jelena Perovanovic, Jyoti Jaswal, Nikola Markovic, Eric Hoffman

**Affiliations:** 1 Center for Genetic Medicine Research Children’s National Medical Center, The George Washington University School of Medicine and Health Sciences, Washington, DC, USA; 2 Department of Civil and Environmental Engineering, University of Maryland College Park, Maryland, USA

## Background

During terminal differentiation of cells, there is typically a transition of the nuclear envelope from the Lamin B protein to Lamin A/C proteins. This is commensurate with exit from the cell cycle, and maintenance of the transcriptional programs associated with the terminally differentiated cells. Dominant missense mutations in Lamin A/C cause a broad spectrum of human genetic disorders, where specific point mutations are associated with defects in specific organs or tissues. We have previously presented a model where Lamin A/C mutations disrupt developmentally appropriate interactions between chromatin and the nuclear envelope and lead to poor coordination of E2F cell cycle pathways and terminal differentiation pathways [1]. One of the phenotypes caused by Lamin A/C mutations is Emery Dreifuss Muscular Dystrophy (EDMD). An X-linked recessive phenocopy of EDMD is caused by loss of function of emerin – a binding partner to Lamin A/C at the nuclear envelope. Here, we tested the hypothesis that emerin plays a role in chromatin remodeling via stabilizing nuclear lamina-heterochromatin interactions necessary for appropriate and time dependent muscle differentiation.

## Material and methods

We used WT and emerin null mouse myogenic stem cells to study transcriptional and epigenetic changes during *in vitro* exit from the cell cycle and differentiation to the myogenic lineage. Specific cell cycle (E2F) and myogenic genes were analyzed by qPCR and ChlP-qPCR to determine mRNA timing and H3K9me3 enrichment on gene promoters. Nuclear lamina-chromatin colocalization was determined and quantified by confocal imaging and Matlab.

## Results

Our results showed that TK1 and other cell cycle genes are inappropriately persistently expressed in emerin null cells during differentiation causing delayed exit from cell cycle. Transcripts marking commitment to the myogenic lineage (myogenin and Mef5A) showed delayed activation on both mRNA and protein level. Epigenetic imprints predicted observed deviations from transcriptional timing in emerin null cells, with persistent suppressive chromatin state on myog promoter upon myogenic induction and failure to appropriately establish repressive histone marks (H3K9me3) on Tk1 promoter (cell cycle). Finally, we showed that the early cell cycle exit and terminal differentiation of emerin null myoblasts were accompanied by decreased H3K9me3 staining at the nuclear periphery (lamin A/C immunostaining).

## Conclusions

Myogenic cells lacking emerin exhibit perturbations in terminal commitment to the myogenic lineage. Our transcriptional, chromatin remodeling and gene promoter accessibility data show that both exit from cell cycle and terminal commitment to myogenesis are disrupted due to inappropriate heterochromatin-nuclear lamina interactions in EMD myogenic cells.
